# An Abbreviated Version of the Digital Trail Making Test‐ Part B

**DOI:** 10.1002/alz70857_104846

**Published:** 2025-12-24

**Authors:** Ileana De Anda‐Duran, Phillip H Hwang, Elizabeth Leverant, Sheina Emrani, Louisa I. Thompson, Stacy L. Andersen, David J. Libon

**Affiliations:** ^1^ Celia Scott Weatherhead Tulane University School of Public Health and Tropical Medicine, New Orleans, LA, USA; ^2^ Department of Epidemiology, Boston University School of Public Health, Boston, Boston, MA, USA; ^3^ New Jersey Institute for Successful Aging, Rowan University, Stratford, NJ, USA; ^4^ University of Pennsylvania, Philadelphia, PA, USA; ^5^ Alpert Medical School of Brown University, Providence, RI, USA; ^6^ Boston University Chobanian & Avedisian School of Medicine, Boston, MA, USA; ^7^ Department of Psychology, Rowan University, Glassboro, NJ, USA; ^8^ Rowan‐Virtua School of Osteopathic Medicine, New Jersey Institute for Successful Aging, Stratford, NJ, USA

## Abstract

**Background:**

With the availability of monoclonal anti‐body treatment for mild cognitive impairment (MCI) and mild dementia due to Alzheimer's disease (AD), there is a need to screen and identify emergent disease as soon as possible. In a companion abstract, a panel of six novel outcome measures from the digital Trail Making Test‐Part B (dTMT‐B) was able to dissociate patients with mild cognitive impairment (MCI) versus normal cognition (NC). The current research fashioned an abbreviated dTMT‐B test and assessed how well this test can dissociate patients with MCI versus NC; and relationships between paper/pencil neuropsychological tests.

**Method:**

Using a digital neuropsychological battery of episodic and working memory tests, memory clinic patients (*n* = 58) were classified into groups presenting with MCI versus NC. An abbreviated dTMT‐B test was created by examining behavior confined to approximately the first half of the test, i.e., target circle ‘1’ through target circle ‘F’. The panel of dTMT‐B process metrics included (1) hit duration or time spent with the pen inside target circles; (2) distance or the length of an imaginary line connecting all targets; and (3) the velocity or the speed pen strokes were drawn.

**Result:**

MCI patients were slightly older and less educated than NC patients; and scored lower on the MMSE. An ANOVA controlled for age, education, and sex found longer hit duration for MCI compared to NC patients (*p* < 0.004, η^2^ =  0.204). No differences were found for total distance or pen stroke velocity. Partial correlations controlled for age, education, and sex found that longer hit duration inside target circles was associated with lower performance on tests measuring attention (WAIS‐IV Digits Forward/ Trails A; r = ‐0.724, *p* < 0.001); working memory (WMS‐IV Symbol Span/ letter fluency; r = ‐0.520, *p* < 0.007); and episodic memory (CVLT‐9 Delay Free recall/ Recognition; r = ‐0.411, *p* < 0.037).

**Conclusion:**

The engineering of the Trail Making Test‐ Part B onto the digital platform has resulted in a panel of new process‐based metrics and potentially increases the versatility of this test. An abbreviated version of the test could easily be deployed to screen for emergent cognitive impairment.

